# Constructing and analysing dynamic models with modelbase v1.2.3: a software update

**DOI:** 10.1186/s12859-021-04122-7

**Published:** 2021-04-20

**Authors:** Marvin van Aalst, Oliver Ebenhöh, Anna Matuszyńska

**Affiliations:** 1 Institute of Quantitative and Theoretical Biology, Heinrich Heine University, Universitätsstr. 1, 40225 Düsseldorf, Germany; 2 CEPLAS - Cluster of Excellence on Plant Sciences, Universitätsstr. 1, 40225 Düsseldorf, Germany

**Keywords:** Research software, Mathematical modelling, ODE, Metabolic networks, Systems biology, Systems medicine, Biomedical systems, Flux analysis, Labelling, Isotope tracing

## Abstract

**Background:**

Computational mathematical models of biological and biomedical systems have been successfully applied to advance our understanding of various regulatory processes, metabolic fluxes, effects of drug therapies, and disease evolution and transmission. Unfortunately, despite community efforts leading to the development of SBML and the BioModels database, many published models have not been fully exploited, largely due to a lack of proper documentation or the dependence on proprietary software. To facilitate the reuse and further development of systems biology and systems medicine models, an open-source toolbox that makes the overall process of model construction more consistent, understandable, transparent, and reproducible is desired.

**Results and discussion:**

We provide an update on the development of modelbase, a free, expandable Python package for constructing and analysing ordinary differential equation-based mathematical models of dynamic systems. It provides intuitive and unified methods to construct and solve these systems. Significantly expanded visualisation methods allow for convenient analysis of the structural and dynamic properties of models. After specifying reaction stoichiometries and rate equations modelbase can automatically assemble the associated system of differential equations. A newly provided library of common kinetic rate laws reduces the repetitiveness of the computer programming code. modelbase is also fully compatible with SBML. Previous versions provided functions for the automatic construction of networks for isotope labelling studies. Now, using user-provided label maps, modelbase v1.2.3 streamlines the expansion of classic models to their isotope-specific versions. Finally, the library of previously published models implemented in modelbase is growing continuously. Ranging from photosynthesis to tumour cell growth to viral infection evolution, all these models are now available in a transparent, reusable and unified format through modelbase.

**Conclusion:**

With this new Python software package, which is written in currently one of the most popular programming languages, the user can develop new models and actively profit from the work of others. modelbase enables reproducing and replicating models in a consistent, tractable and expandable manner. Moreover, the expansion of models to their isotopic label-specific versions enables simulating label propagation, thus providing quantitative information regarding network topology and metabolic fluxes.

**Supplementary Information:**

The online version contains supplementary material available at 10.1186/s12859-021-04122-7.

## Background

Mathematical models are accepted as valuable tools for advancing biological and medical research [1, 2]. In particular, models based on ordinary differential equations (ODEs) have found application in a variety of fields. Most recently, deterministic models simulating the dynamics of infectious diseases gained the interest of the general public during the Covid-19 pandemic. Consequently, a large number of ODE based mathematical models were developed and discussed, even in nonscientific journals [3–5]. Such focus on mathematical modelling is not surprising, because computational models allow for methodical investigations of complex systems under fixed, controlled and reproducible conditions. Hence, the effect of various perturbations of the systems can be inspected systematically in silico.

The model building process itself plays an important role in integrating and systematising vast amounts of available information [6]. Properly designed and verified computational models serve various purposes. They are used to develop hypotheses to guide the design of new research experiments (e.g., in immunology to study lymphoid tissue formation [7]). Models can also support metabolic engineering efforts (e.g., identification of enzymes to enhance essential oil production in peppermint [8]). More recently, models contribute to tailoring medical treatment to individual patient in the spirit of precision medicine (e.g., in oncology [2]). Finally, modelling results guide political decision making and governmental strategies (see the review on the impact of modelling for European Union Policy [9]). Considering their potential impact, models must be openly accessible so that they can be verified and corrected, if necessary.

In many publications, modelling efforts are justified by the emergence of extraordinary amounts of data provided by new experimental techniques. However, arguing for the necessity of model construction only because a certain type or amount of data exists, ignores several important aspects. Computational models are generally a result of months, if not years, of intense research, which involves gathering and sorting information, simplifying numerous details and distilling out the essentials, implementing the mathematical description in computer code, carrying out performance tests, and, finally, validating the simulation results. Our understanding of many phenomena could be deepened if, instead of constructing yet another first-generation model, we could efficiently build on the knowledge that was systematically collected in previously developed models. Moreover, the knowledge generated during the model construction process is often lost, e.g. because the main developer left the research team.

Preservation of the information collected in the form of a computational model has become an important quest in systems biology, and has, to some extent, been addressed by the community. Development of the Systems Biology Markup Language (SBML) [10] for unified communication and storage of biomedical computational models, and the existence of the BioModels repository [11] already ensures the survival of models beyond the academic life of their developers, or the lifetime of the software used to create them. However, a model in SBML format rarely reveals the logic of model construction. The structure of modelbase code promotes consistent and transparent description of the model components (such as reaction rates), hence the logic of construction becomes inherently clear. Such knowledge loss can be prevented by providing simple-to-use toolboxes that enforce a universally readable model construction format.

For these reasons we developed modelbase[12], a Python package that allows the user to easily document the model building process. On the one hand, we defined the core of the model construction process, while on the other hand, the software does not make these definitions too strict, and fully integrates the model construction process into the Python programming language. This differentiates modelbase from many other Python-based modelling tools (such as ScrumPy [13], PySCeS [14], PySB [15] or tellurium [16, 17]) and other mathematical modelling languages (recently reviewed from a software engineering perspective by Schölzel and colleagues [18]). We would in particular like to stress a fundamental difference in the philosophy of modelbase, which distinguishes it from the other Python-based tools. In ScrumPy, PySCeS and tellurium, models are objects that are constructed by either SBML import or by a human-readable string (e.g. the Antimony representation chosen in tellurium [17]), which have methods for their numeric simulations and analysis. However, once constructed, the objects are not designed to be further modified. A modular design of different, but similar models, which all depend on sets of analogous modules, is thus difficult to represent. PySB aims at providing systematic construction methods, adding e.g. ’monomers’ and ’rules’ how these are converted. However, PySB deliberately ignores and overrides standard Python behaviour, making it difficult to keep multiple models in one namespace. In modelbase, models and simulations are two different types of objects. In analogy to experiments, a model corresponds to the biological entity, such as a cell, whereas a simulation corresponds to a particular experiment that is performed on the entity. A model object can be arbitrarily modified by numerous methods. Typically, a model is systematically constructed by instantiating an empty model object, to which components are added by dedicated methods. In this way, the model construction process remains maximally transparent, is fully integrated into the Python programming language, and is completely reproducible. Flexibility to modify and alter the model structure (incl. parameters) is ensured in this way.

Here we report new features in modelbase v1.2.3, developed over the last two years. We have significantly improved the interface to make model construction easier and more intuitive. The accompanying repository of replicated, published models, available from our GitLab project, has been considerably expanded, and now includes a diverse selection of biomedical models (see Additional file 1: Table 2). This diversity highlights the general applicability of our software. Essentially, every dynamic process that can be described by a system of ODEs can be implemented with modelbase.

## Implementation

modelbase is a Python package to facilitate construction and analysis of ODE based mathematical models of biological systems. Version 1.2.3 introduces changes not compatible with the previous official release, version 0.2.5 [12]. All API changes are summarised in the official documentation hosted by ReadTheDocs and the differences between the versions are summarised in Table 1.

**Table 1:** Key changes between the first published version of modelbase [12] and the current update

Functionality	modelbase 0.2.5	modelbase 1.2.3
Initialization	Model takes only parameters as an argument	Model takes as arguments all other model components as dictionaries
Parameters	Hidden as a private attribute	Replaced with a vanilla dictionary
Derived parameters	No function to calculate from other model parameters	Called on initialization and prior to any numerical operations
Handling of time-dependent reactions	Time given in kwargs	Modifiers argument is introduced
Simulation	Integration via timeCourse that takes an array of time points for Simulation	Integration via simulate that takes only the endpoint of the simulation, default starting point t = 0, otherwise starts where the last simulation ended
Labelling features	Focused on carbon labelling problems	Reference of the word carbon was changed to label
Method to calculate relative label distribution in steady-state	None available	Via LinearLabelModel
Scan steady-state concentrations depending on parameter values	None available	Via parameter_scan
SBML support	Export of model stoichiometries	Import of models that match the capabilities of modelbase and full export of models using ratelaw
Metabolic Control Analysis support	None available	A full suite of methods to calculate and plot elasticities via mca module
Predefined kinetic laws	None available	Via ratelaw module
Plotting support	Time course plots	Phase-plane analysis

The model building process starts by creating a modelling object of the dedicated Python class Model and adding to it the chemical compounds of the system. Then, following the intuition of connecting the compounds, the reaction network is constructed by adding the reactions one by one. Each reaction requires stoichiometric coefficients and a kinetic rate law. The latter can be provided either as a custom function or by selecting one from the newly provided library of rate laws. The usage of this library (ratelaws) reduces the repetitiveness by avoiding boilerplate code. It requires the user to explicitly define reaction properties, such as directionality. This contributes to a systematic and understandable construction process, following the second guideline from the Zen of Python, the guiding principles for Python’s design^1^: “Explicit is better than implicit”.

From this, modelbase automatically assembles the system of ODEs. It also provides numerous methods to conveniently retrieve information about the model. In particular, the get_* methods can be used to inspect all the components of the model, and calculate reaction rates for given concentration values. These functions have multiple variants that return all the common data structures (array [19], dictionary, data frames [20]).

**Fig. 1:**
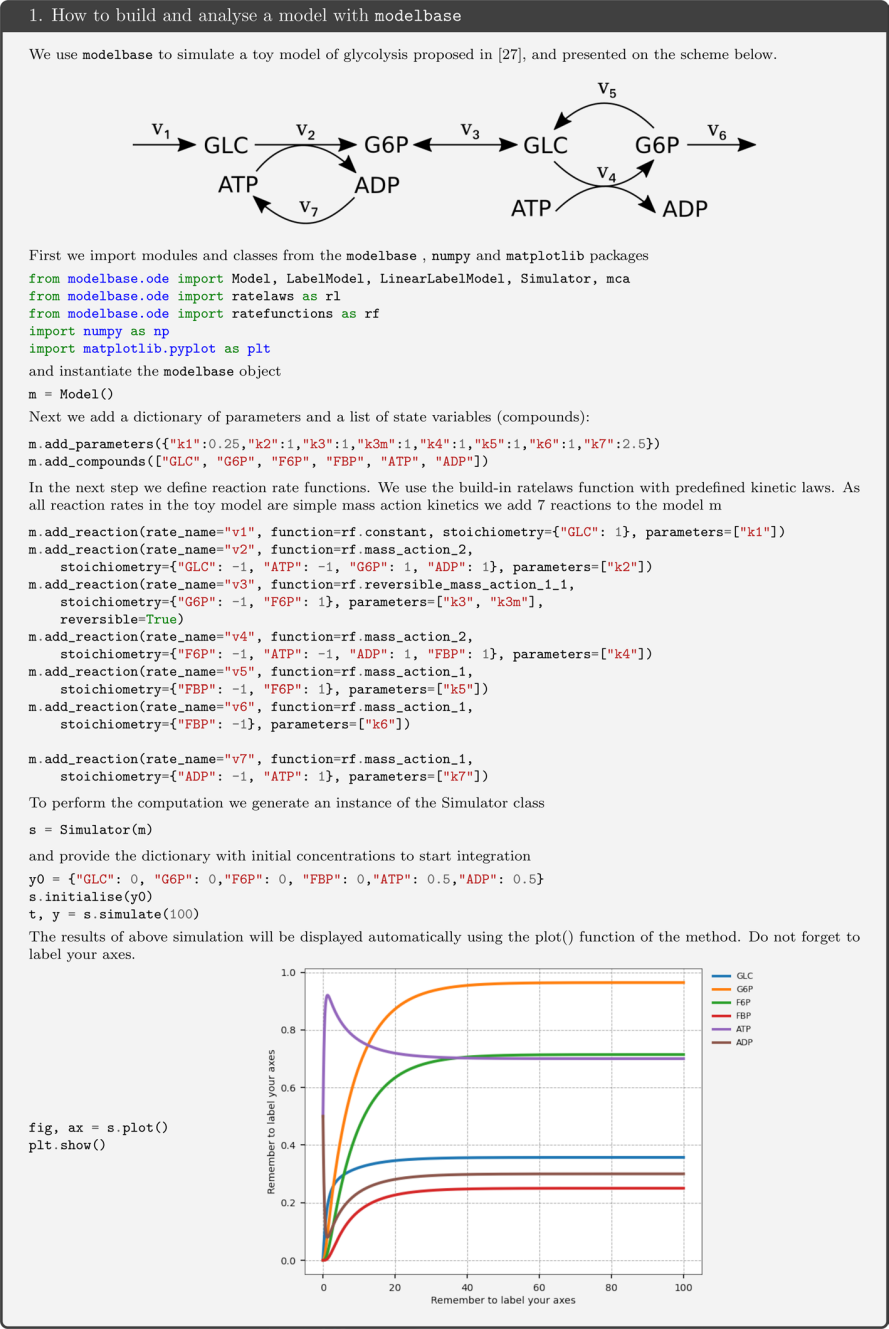
An example of how to build and analyse a model with modelbase

After the model building process is completed, simulation and analyses of the model are performed with the Simulator class. Currently, we offer interfaces to two integrators to solve stiff and non-stiff ODE systems. Provided the Assimulo package [21] is available, as recommended in our installation guide, modelbase will use CVode, a variable-order, variable-step multi-step algorithm. The CVode class provides a direct connection to Sundials [22] which is a powerful industrial solver and robust time integrator, with a high computing performance. If Assimulo is not available, modelbase will automatically use the SciPy library [23]. Specifically lsoda will be used to integrate the model, which in our experience resulted in lower computing performance [24]. The whole process of assembling a model has been summarised in Fig. 1.

### Metabolic control analysis

**Fig. 2:**
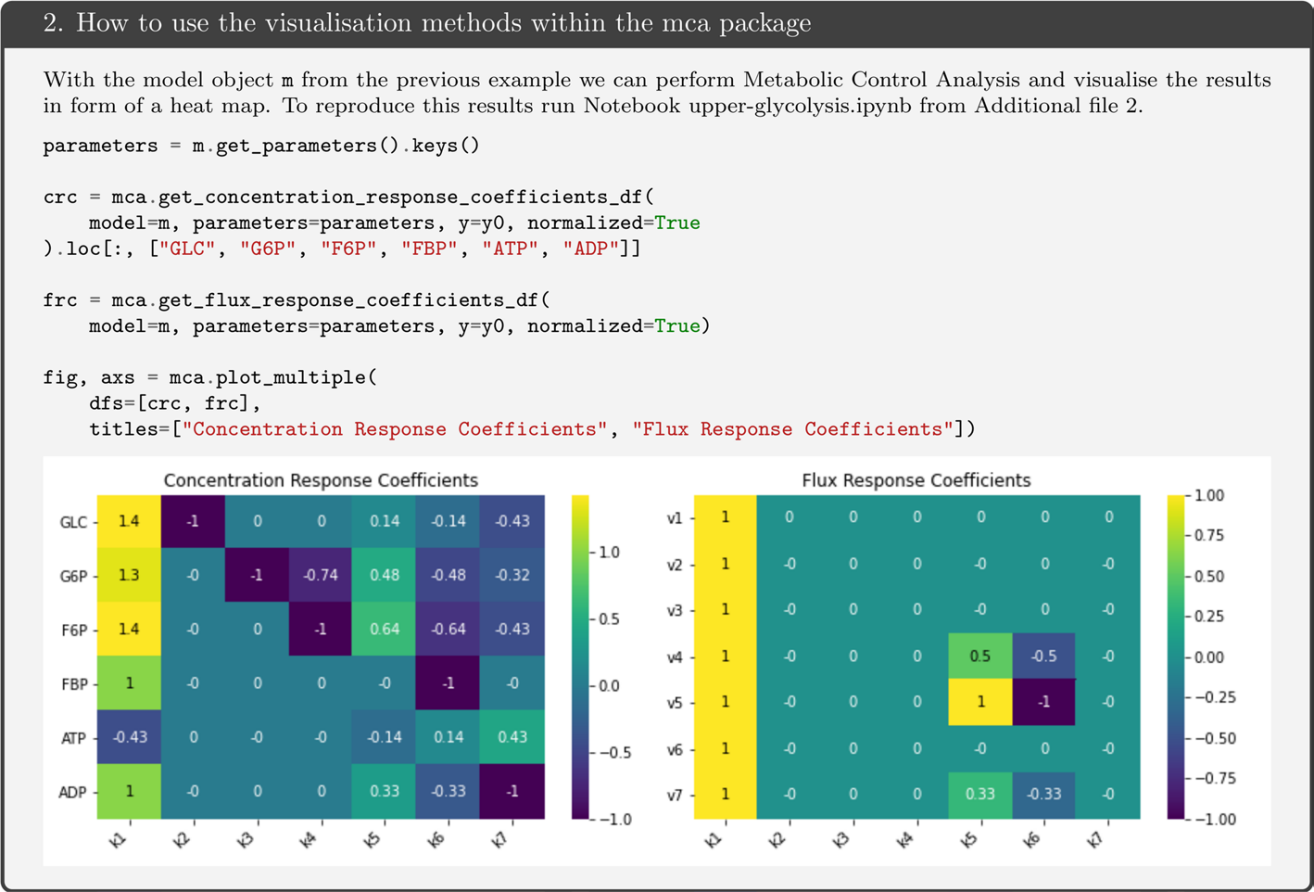
An example of how to use the visualisation methods within the mca package

Sensitivity analysis provides a theoretical foundation to systematically quantify the effects of small parameter perturbations on global system behaviour. In particular, Metabolic Control Analysis (MCA), initially developed to study metabolic systems, is an important and widely used framework providing quantitative information about the response of the system to perturbations [25, 26]. The new version of modelbase has a full suite of methods to calculate elasticities. These can be plotted as a heat-map, giving a clear and intuitive colour-coded visualisation of the results. An example of such visualisation, for a re-implemented toy model of the upper part of glycolysis (Section 3.1.2 [27]), can be found in Fig.  2.

### Visualisation support

Many of the existing software packages for building computational models restrict the users by providing unmodifiable plotting routines with predefined settings that may not suit their personal preferences. In modelbase v1.2.3 plotting functions allow the user to pass optional keyword-arguments (often abbreviated as **kwargs), similar to Tellurium [17]. All plot elements are accessible and available for change, providing a transparent and flexible interface to the commonly used matplotlib library [28]. The easy access functions that visualise the results of simulations were expanded from the previous version. They now include funcitonality to plot selections of compounds or fluxes, phase-plane analysis and the results of MCA. An example of the latter is included in Fig. 2.

### Models for isotope tracing

modelbase has also been developed to aid the in silico analyses of label propagation during isotopic studies. To simulate the dynamic distribution of isotopes, all possible labelling patterns for all intermediates need to be created. By providing an atom transition map in the form of a list or a tuple, all $2^N$ isotope-specific versions of a chemical compound are created automatically, where *N* denotes the number of possibly labelled atoms. Changing the name of the previous function carbonmap to labelmap in v1.2.3 acknowledges the diversity of possible labelling experiments that can be reproduced with models built using our software.

#### Isotope tracing under stationary conditions

**Fig. 3:**
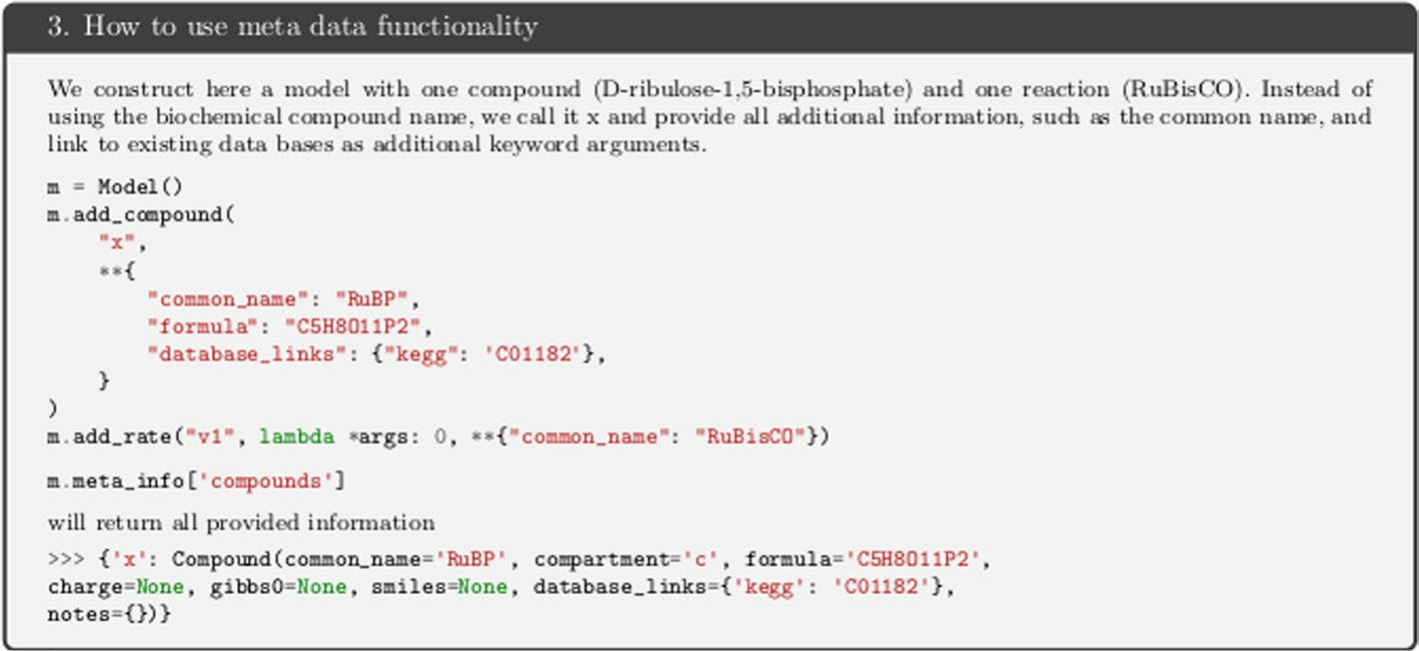
An example of how to use metadata functionality

Sokol and Portais derived the theory of dynamic label propagation under the stationary assumption [29]. In steady-state, the space of possible solutions is reduced and the labelling dynamics can be represented by a set of linear differential equations. We have used this theory and implemented an additional class LinearLabelModel that allows rapid calculation of the label propagation given the steady-state concentrations and fluxes of the metabolites [29]. modelbase can automatically build the linear label model from user provided label maps. An example of such a model is provided in Fig. 3, where we simulate label propagation in a linear non-reversible pathway, see Fig. 1 in [29] for comparison. The linear label models are constructed using modelbase  rate laws, and hence can be fully exported as an SBML file.

### Model metadata

**Fig. 4:**
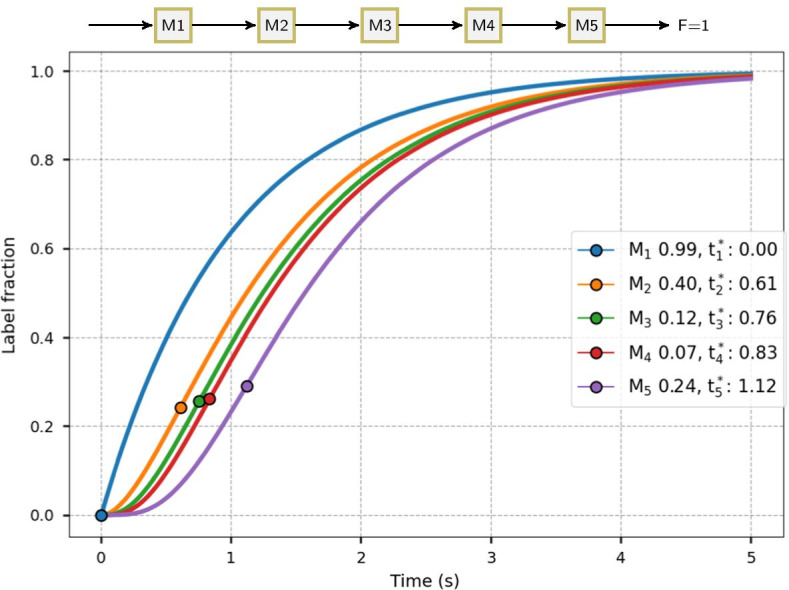
Labelling curves in a linear non-reversible pathway. Example of label propagation curves for a linear non-reversible pathway of five randomly sized metabolite pools, as proposed in the paper by Sokol and Portais [29]. Circles mark the position at which the first derivative of each labelling curve reaches maximum. In the original paper, this information has been used to analyse the label shock wave (LSW) propagation. To reproduce these results run the label-propagation-2015.ipynb Jupyter Notebook from the Additional file 4: Jupyter Notebook label-propagation-2015.ipynb

Many models lose their readability due to the inconsistent, intractable or misguided naming conventions. An example is a model with reactions named v1-v10, without referencing them properly. By providing metadata fields for all modelbase objects, the user can abbreviate component names in a personally meaningful manner and supply additional annotation information in accordance with recognised standards, such as MIRIAM [30]. An example of how to use metadata functionality is provided in Fig. 4. This interface can also be used to supply additional information, such as the unit of a parameter.

### SBML support

In contrast to the previous modelbase version, where we only supported the export of stoichiometric models to SBML format, we now support both import and export of kinetic models. The full summary of the SBML concepts supported by modelbase is documented in the official SBML test suite, where the output of our tests is stored. Examples where SBML models are imported and exported, using our build_model_from_sbml and write_sbml_model functions, are supplied in the modelbase  documentation.

## Results and discussion

With the newly implemented changes, modelbase is more versatile and user friendly. As argued before, its strength lies in its flexibility and applicability to virtually any biological system with dynamics that can be described using an ODE system. There exist countless mathematical models of biological and biomedical systems derived using ODEs. Many of these models are rarely re-used, at least not to the extent that could be reached, if models were shared in a readable, understandable and reusable way [18]. Our package can be used efficiently both for the development of new models, as well as the reconstruction of existing ones. We are confident that modelbase will in particular support users with limited modelling experience in re-constructing already published work, serving as a starting point for their further exploration and development. We have previously demonstrated the versatility of modelbase by re-implementing mathematical models previously published without the source code: two models of biochemical processes in plants [31, 32], and a model of the non-oxidative pentose phosphate pathway of human erythrocytes [33, 34]. To present how the software can be applied to study medical systems, we used modelbase to re-implement various models, not published by our group, and reproduced key results of the original manuscripts. It was beyond our focus to verify the scientific accuracy of the corresponding model assumptions. We selected these examples to show that despite describing different processes, they all share a unified construct. This highlights that by learning how to build a dynamic model with modelbase, the user do not learn how to build a one-purpose model, but in fact expands the toolbox to be capable of replicating any given ODE based model. All examples are available as Jupyter notebooks and listed in the Additional file 3: Jupyter Notebook upper-glycolysis.ipynb.

### Compartment model for disease evolution

**Fig. 5:**
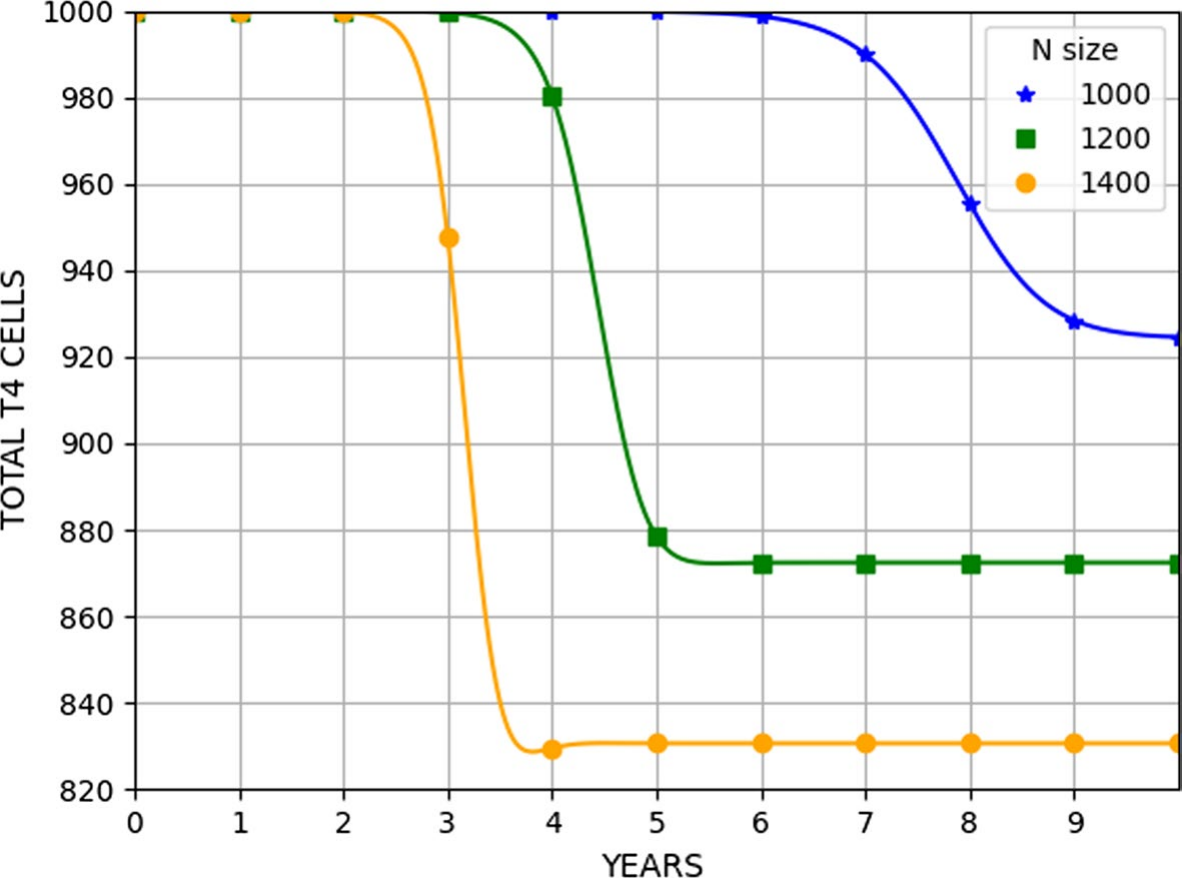
The total CD4+ T-cell population versus time after the infection. We have reproduced the results from Fig. 4 from the original paper [35] showing the decrease in the overall population of CD4+ T-cell (uninfected + latently infected + actively infected CD4+) over time, depending on the number of infectious particles produced per actively infected cell (N). To reproduce these results run the hiv-t4cell.ipynb Jupyter Notebook from the Additional file 5: Jupyter Notebook hiv-t4cell.ipynb

For this paper, we surveyed available computational models and selected a relatively old publication of significant impact, that was published without providing the computational source code, nor details regarding the numerical integration. We chose a four-compartment model of HIV immunology that investigates the interaction of a single virus population with the immune system described only by the CD4$^+$ T cells, commonly known as T helper cells [35]. We implemented the four ODEs describing the dynamics of uninfected (T), latently infected (L), actively infected CD4$^+$ T cells (A), and infectious HIV population (V). In Fig. 5, we reproduce the results from Fig. 4 in the original paper, whereby changing the number of infectious particles produced per actively infected cell (N) we follow the dynamics of the overall T cell population (T+L+A) over a period of 10 years. The model was also used to explore the effect of azidothymidine, an antiretroviral medication, by decreasing the value of N after 3 years by 25% or 75%, mimicking the blocking of the viral replication of HIV. A more detailed description of the time-dependent drug concentration in the body is often achieved with pharmacokinetic models. Mathematical models based on a system of differential equations that link the dosing regimen with the dynamics of a disease are called pharmacokinetic-pharmacodynamic (PK-PD) models [36]. The next example explores how modelbase can be used to develop such models.

### PK-PD models and precision medicine

**Fig. 6:**
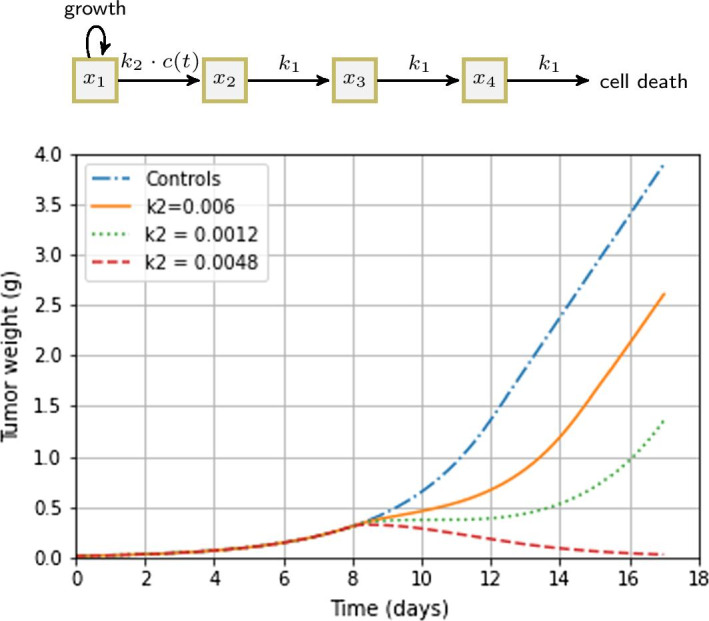
Compartmental pharmacokinetic-pharmacodynamic model of tumour growth after anticancer therapy. We have reproduced the simplified version of the PK-PD model of tumour growth, where the PK part is reduced to a single input and simulated the effect of drug potency ($k_2$) on tumour growth curves. The system of four ODEs describing the dynamics of the system visualised on a scheme above is integrated over a period of 18 days. We systematically changed the value of $k_2$, assuming a single drug administration on Day 9. We have obtained the same results as in Fig. 4 in the original paper [40]. To reproduce these results run the tumour-growth-2004.ipynb Jupyter Notebook from the Additional file 6: Jupyter Notebook tumour-growth-2004.ipynb

Technological advances forced a paradigm shift in many fields, including medicine, making more personalised healthcare not only a possibility but a necessity. A pivotal role in the success of precision medicine will be to correctly determine dosing regimes for drugs [37]. PK-PD models provide a quantitative tool to support this [38]. PK-PD models have proven successful in many fields, including oncology [39], here we use the classical tumour growth model developed by Simeoni and colleagues, originally implemented using the industry-standard software WinNonlin [40]. As the full pharmacokinetic model is not fully described, we reproduced only the highly simplified case, where we assume a single drug administration and investigate the effect of drug potency ($k_2$) on simulated tumour growth curves. In Fig. 6 we plot the simulation results of the modelbase implementation of the system of four ODEs over a period of 18 days, where we systematically changed the value of $k_2$, assuming a single drug administration on Day 9. With the MCA suite available in our software, we can calculate the response to perturbation of all other system parameters. Such a quantitative description of the system’s dynamics to local parameter perturbation provides support for further studies of the rational design of combined drug therapy and the discovery of new drug targets, as described in the review by Cascante and colleagues [41].

### Modelling of infectious diseases with SIR models

**Fig. 7:**
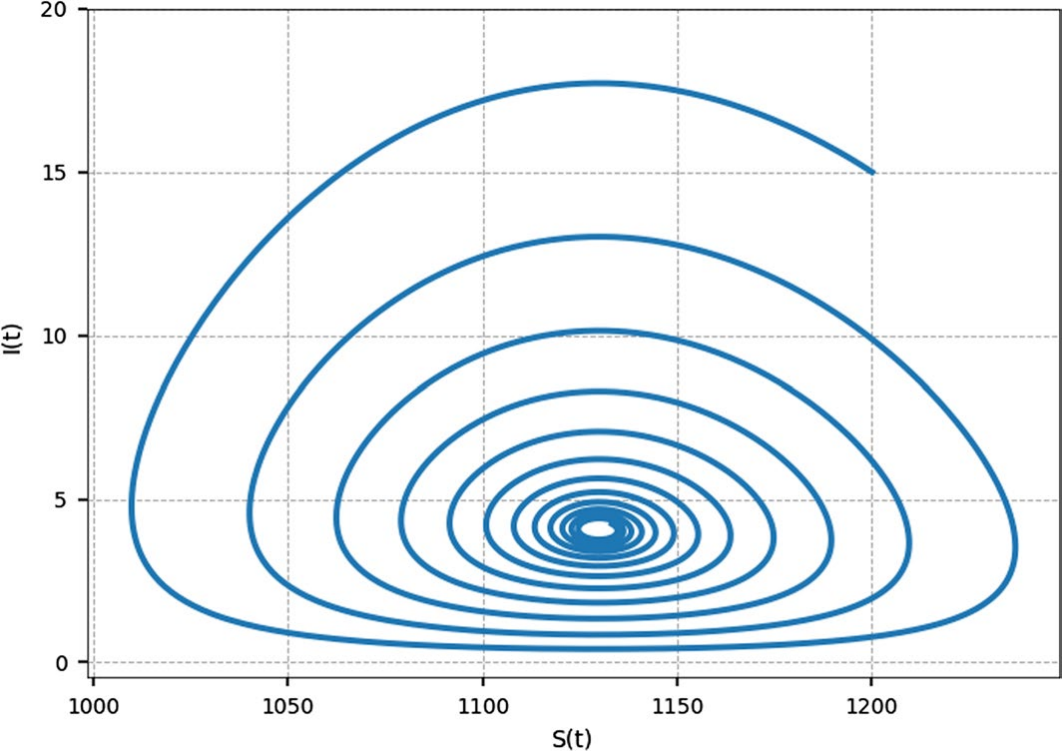
Sample phase portrait obtained with SIR model with oscillations. SIR model with vital dynamics including birth rate has been adapted based on the autonomous model to simulate periodicity of chickenpox outbreak in Hida, Japan [45]. To reproduce these results run the sir-model.ipynb Jupyter Notebook from the Additional file 7: Jupyter Notebook sir-model.ipynb

Finally, compartmental models based on ODE systems have a long history of application in mathematical epidemiology [42]. Many of them, including numerous recent publications studying the spread of coronavirus, are based on the classic epidemic Susceptible-Infected-Recovered (SIR) model, originating from the theories developed by Kermack and McKendrick at the beginning of the last century [43]. One of the most important insights gained from simulating the dynamics of infectious disease is the existence of disease-free or endemic equilibrium, and assessment of its stability [44]. Interestingly, periodic oscillations have been observed for several infectious diseases, including measles, influenza and smallpox [42]. To provide an overview of more modelbase functionalities we have implemented a relatively simple SIR model based on the recently published autonomous model for smallpox [45]. We have generated damped oscillations and visualised them using the built-in function plot_phase_plane (Fig. 7). In the accompanying Jupyter notebook we demonstrate using modelbase, how simply the SIR model can be built and how to modify it to construct more variants, such as the SEIR (E-exposed) or SIRD (D-deceased) models.

## Conclusions

Here, we are presenting an update of our modelling software that simplifies the process of building mathematical models based on ODEs. modelbase  is fully embedded in the Python programming language. It facilities a systematic construction of new models, and replication of models in a consistent, tractable and expandable manner. As ODEs are a core method to describe the dynamical systems, we hope that our software will serve as the base for deterministic modelling, hence its name. With the smoothed interface and clearer description of how the software can be used for medical purposes, such as simulation of possible drug regimens for precision medicine, we expect to broaden our user community. We envisage that by providing the MCA functionality, users new to mathematical modelling will adopt a working scheme where such sensitivity analyses are an integral part of model development and analysis. The value of sensitivity analyses is demonstrated by considering how the results of such analyses have given rise to new potential targets for drug discovery [41]. We anticipate that the capability of modelbase to automatically generate isotopic label-specific models will prove useful in predicting fluxes and label propagation dynamics through various metabolic networks. In emerging fields such as computational oncology, such models will be useful to, e.g., predict the appearance of labels in cancer cells.

## Availability and requirements

*Project name*: modelbase

*Project home page*: https://pypi.org/project/modelbase/

*Code repository*: https://gitlab.com/qtb-hhu/modelbase-software

*Version published*: 1.2.3

*Date published*: 14 Jan 2021

*Documentation*: https://modelbase.readthedocs.io/en/latest

*Operating system(s)*: Platform independent

*Programming language*: Python

*Other requirements*: None

*Licence*: GNU General Public License (GPL), version 3

*Any restrictions to use by non-academics*: None.

## Supplementary Information

**Additional file 1**. Table with the list of available models in the repository

**Additional file 2**. README.md with the Installation instructions

**Additional file 3**. Jupyter Notebook upper-glycolysis.ipynb: toy model of the upper path of glycolysis asintroduced in [27]

**Additional file 4**. Jupyter Notebook label-propagation-2015.ipynb: an example of a linear non-reversible pathwayof five randomly sized metabolites and label propagation experiments, as proposed in the paper by Sokol and Portais [29]

**Additional file 5**. Jupyter Notebook hiv-t4cell.ipynb: a model of the dynamics of HIV infection of CD4+ cells,considering three populations of T cells and free virus, proposed by Perelson, Kirschner and de Boer [35]

**Additional file 6**. Jupyter Notebook tumour-growth-2004.ipynb: minimal pharmacokinetic-pharmacodynamic(PK-PD)model linking that linking the dosing regimen of an anticancer agent to the tumour growth, proposed bySimeoni and colleagues [40]

**Additional file 7**. Jupyter Notebook sir-model.ipynb: classic epidemic Susceptible-Infected-Recovered (SIR) modelparameterised as the autonomous model used to simulate periodicity of chickenpox outbreak in Hida, Japan [45]

## Data Availability

All data generated or analysed during this study are included in this published article. Additionally, code documentation can be found here https://modelbase.readthedocs.io/en/latest and more Jupyter Notebooks with teaching materials and models here https://gitlab.com/qtb-hhu/models. If you have any questions regarding modelbase, you are very welcome to ask them on our issues page or by contacting any of the authors. It is our mission to enable reproducible science and to help to put the theory into action.

## Abbreviations

MCA: Metabolic control analysis
ODE: Ordinary differential equations
PK-PD: Pharmacokinetic-pharmacodynamic
SBML: Systems biology markup language

## Footnotes

1 python-c “import this”.
