# Supplementary figures and images for: Constructing and analysing dynamic models with modelbase v1.2.3: a software update
Source: BMC Bioinformatics. 2021 Apr 20;22:203. doi: 10.1186/s12859-021-04122-7 (PMC8056244; doi:10.1186/s12859-021-04122-7)

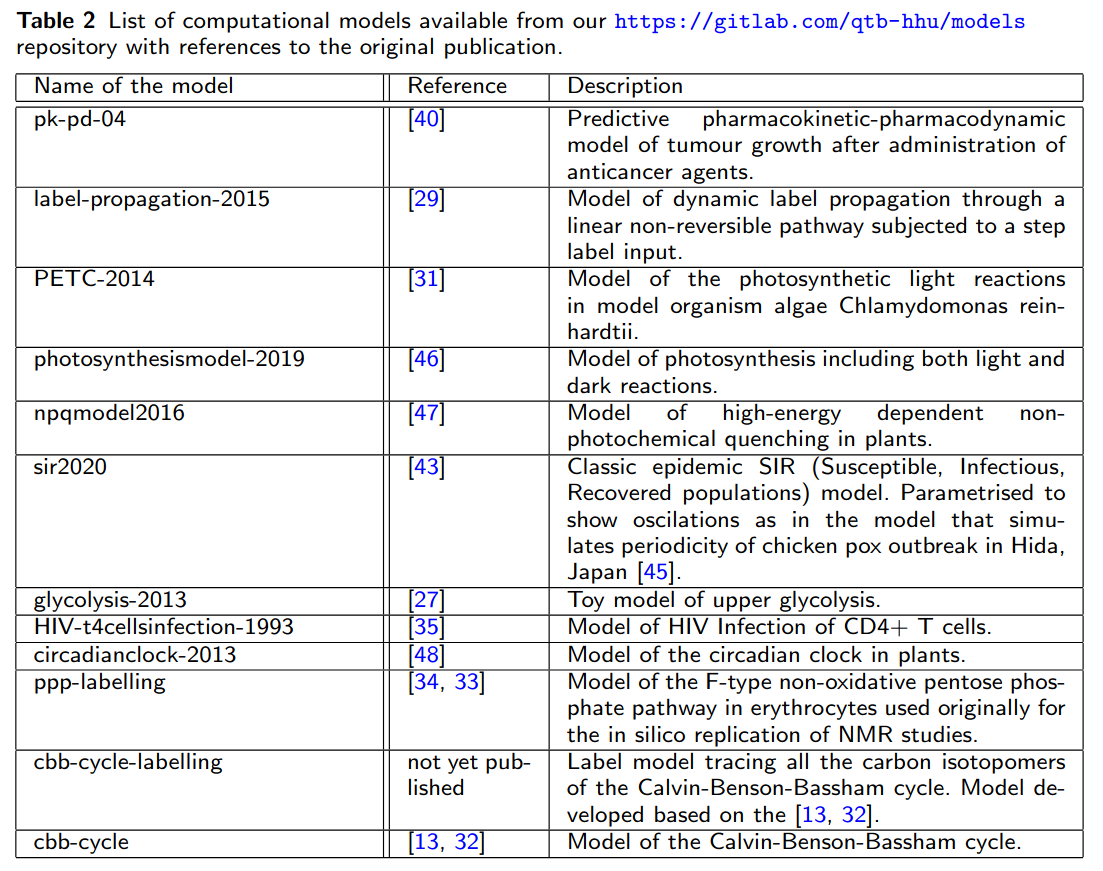

Supplement: Supplementary file 1 — Additional file 1. Table with the list of available models in the repository [file 12859_2021_4122_MOESM1_ESM.pdf]
